# Assessment of *Acropora palmata* in the Mesoamerican Reef System

**DOI:** 10.1371/journal.pone.0096140

**Published:** 2014-04-24

**Authors:** Rosa E. Rodríguez-Martínez, Anastazia T. Banaszak, Melanie D. McField, Aurora U. Beltrán-Torres, Lorenzo Álvarez-Filip

**Affiliations:** 1 Unidad Académica de Sistemas Arrecifales, Instituto de Ciencias del Mar y Limnología, Universidad Nacional Autónoma de México, Puerto Morelos, Quintana Roo, México; 2 Healthy Reefs Initiative, Belize City, Belize; California Polytechnic State University, United States of America

## Abstract

The once-dominant shallow reef-building coral *Acropora palmata* has suffered drastic geographical declines in the wider Caribbean from a disease epidemic that began in the late 1970s. At present there is a lack of quantitative data to determine whether this species is recovering over large spatial scales. Here, we use quantitative surveys conducted in 107 shallow-water reef sites between 2010 and 2012 to investigate the current distribution and abundance of *A. palmata* along the Mesoamerican Reef System (MRS). Using historical data we also explored how the distribution and abundance of this species has changed in the northern portion of the MRS between 1985 and 2010–2012. *A. palmata* was recorded in only a fifth of the surveyed reef sites in 2010–2012. In the majority of these reef sites the presence of *A. palmata* was patchy and rare. Only one site (Limones reef), in the northernmost portion of the MRS, presented considerably high *A. palmata* cover (mean: 34.7%, SD: 24.5%). At this site, the size-frequency distribution of *A. palmata* colonies was skewed towards small colony sizes; 84% of the colonies were healthy, however disease prevalence increased with colony size. A comparison with historical data showed that in the northern portion of the MRS, in 1985, *A. palmata* occurred in 74% of the 31 surveyed sites and had a mean cover of 7.7% (SD = 9.0), whereas in 2010–2012 this species was recorded in 48% of the sites with a mean cover of 2.9% (SD = 7.5). *A. palmata* populations along the MRS are failing to recover the distribution and abundance they had prior to the 1980s. Investigating the biological (e.g., population genetics) and environmental conditions (e.g., sources of stress) of the few standing reefs with relatively high *A. palmata* cover is crucial for the development of informed restoration models for this species.

## Introduction

Historically, *Acropora palmata* (Lamarck, 1816) was a dominant reef-building coral in wave-exposed and high-surge reef zones, typically at depths less than 10 m, both in terms of its abundance and its contribution to reef accretion rates [1]–[6]. Its branching morphology, high growth rate, asexual reproduction through fragmentation and efficient recuperation from lesions, allowed this species to recover relatively quickly after the impact of hurricanes and other physical stressors and made it a successful competitor as well as a functionally important component of Caribbean reefs [7]. This once-abundant and architecturally complex species provided habitat to many species [8], [9] and served as a natural barrier that dissipated wave and current energy reaching other coastal ecosystems [7].

In the 1970s and 1980s, *A. palmata* underwent severe declines in its abundance and distribution from the outbreak of white-band disease, which killed the coral tissue, and the subsequent impact of severe hurricanes that broke down the coral skeletons [5], [7], [10]–[13]. Populations of *A. palmata*, and other coral species, then experienced increasingly unfavorable conditions, such as the appearance of white-pox disease, anomalously high sea surface temperatures, and increasing levels of eutrophication and pollution, that exacerbated coral mortality [7], [14]–[16]. Although, it has been estimated that between 80% and 98% of *A. palmata* individuals have been lost in the Caribbean in the last three decades [7], [17], there is only a handful of published studies on the quantitative changes in the distribution and abundance (density and cover data) of this species over time [18]–[20]. The decline of acroporids has profound consequences for the functioning and structure of Caribbean reefs as no other reef-building species combines such a complex branching morphology and high calcification rates [21]. The mortality of *A. palmata* represents a substantial loss in the rates of carbonate production, ultimately impairing reef growth and leading to net erosion [22]–[24]. In addition, the mortality of *A. palmata* colonies and subsequent erosion of their remnant skeletons represent a considerable reduction in spatial heterogeneity of Caribbean reefs that may drive declines in biodiversity, compromise fisheries productivity and reduce coastal protection from wave energy [9]. *A. palmata* is now listed as a critically endangered species under the International Union for Conservation of Nature Red List [17], [25], it is listed in the Convention on International Trade in Endangered Species (CITES) Appendix II, it has been listed as threatened by the national government of Mexico [26] and has been proposed for listing as endangered under the US Endangered Species Act [27].

The Mesoamerican Reef System (MRS) is recognized as one of the most biodiverse regions in the wider Caribbean, and reef-based tourism is a major contributor to the local economies of Mexico, Belize, Guatemala and Honduras [28]–[30]. In addition, coral reefs in this region support commercial and subsistence fishing, and provide important ecosystem services such as coastal protection to urban and tourist developments [28]. The signing of the Tulum Declaration in 1997 represented the start of a multinational effort to promote the sustainable use of MRS resources and the establishment of a network of marine protected areas (MPAs) to protect key coastal ecosystems [28]. Several scientific studies, monitoring programs and local *A. palmata* rehabilitation projects have been conducted in the MRS since that time [22], [30]. However, and despite all of these efforts, little is known about the current status of *A. palmata* in the MRS. To our knowledge there are no reports of the recovery of this species in the MRS comparable to those reported for other Caribbean reefs, such as Haulover Bay in St. John, US Virgin Islands [16] and Los Roques, Venezuela [31].

Here we assess the current distribution and abundance of *Acropora palmata* in the Mesoamerican Reef System, by using a region-wide database compiled by the Healthy Reef Initiative (HRI) between 2010 and 2012. In addition, to explore how the relative cover and distribution of *A. palmata* has changed over the last two decades for a portion of this system, we compare the data obtained in 2010–2012 with data generated in 1985 in the northern portion of the system (Mexican Caribbean).

## Methods

### Mesoamerican Reef System Regional Survey (2010–2012)

This region-wide dataset is part of the monitoring program of the Healthy Reefs Initiative (HRI) and the Atlantic and Gulf Reef Rapid Assessment program (AGRRA), which aims to develop measurable ranking criteria for indicators of the ecological condition of the Mesoamerican Reef [29], [32]. The site-selection protocol was based on the benthic habitat maps produced by the Millennium Reef Mapping Program [33], and consisted of randomly selected reef sites using geographical information systems. Equally-sized spatial units, represented by geometrical forms (hexagons), were used to stratify the MRS reef layer based on geomorphic characteristics and depth, and included the following reef habitats: patch reefs, bank reefs and the reef crest and fore-reef zones of fringing reefs [32], [34]. Spatial units were then randomly selected by region and country [32]. A reef site is thus defined as a more or less homogeneous habitat, roughly 200 m×200 m in spatial extent, situated in a geomorphic zone of a reef on an insular or continental shelf [32]. Although sites were selected to represent the whole area within the MRS [29], [32], due to logistical reasons it was not possible to survey some portions of the reef system (*e.g.,* the southern part of the Mexican Caribbean). Given that our specific interest was to represent the potential habitats for *A. palmata*, we only used the information for reef sites at ≤10 m depth.

**Figure 1:**
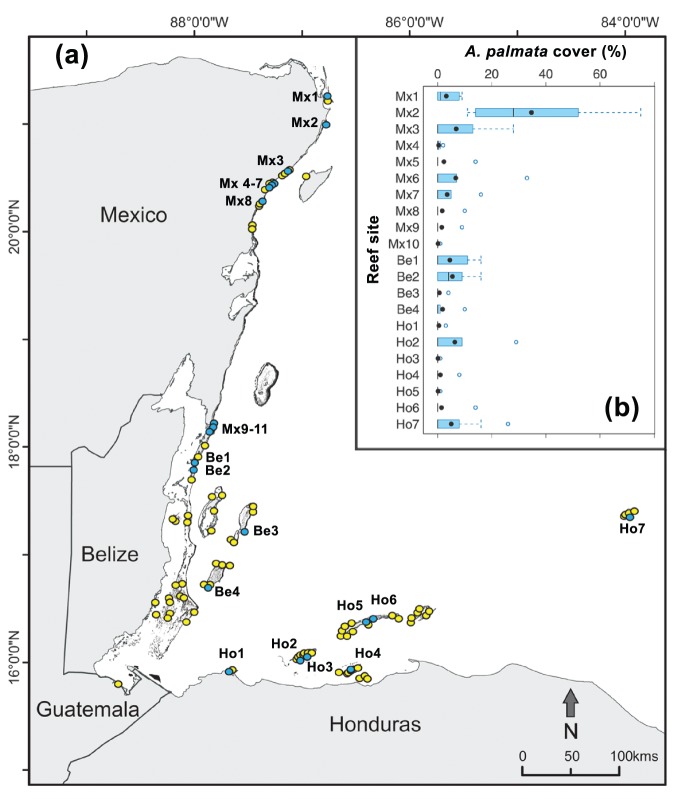
Distribution and abundance of *Acropora palmata* in the Mesoamerican Reef System. (**a**) Location of the 107 coral reef sites sampled between 2010 and 2012. Blue circles represent sites in which *Acropora palmata* was recorded in the transects. Yellow circles represent sites where *A. palmata* was not recorded. (**b**) Box-and-whisker plot of percent *A. palmata* cover at the 21 sites where this species was found along the Mesoamerican Reef System in 2010–2012. The bottom and top of the box are the first and third quartiles, respectively, the black dot inside the box is the average and the black line is the median. Whiskers are the lowest datum still within 1.5 times that of the lower quartile and the highest datum still within 1.5 times that of the upper quartile. The open circles at the end of the boxplot represent outliers (transects with *A. palmata* cover values 1.5 times less or greater than the interquartile range). Sites are arranged from north to south for Mexico and Belize and from West to East for Honduras. For site codes see Table S1.

In total, for the regional assessment of *Acropora palmata* along the MRS we analyzed data collected at 107 shallow-water (≤10 m) reef sites (23 in Mexico, 36 in Belize, 1 in Guatemala and 47 in Honduras; Table 1; Fig. 1a). Each reef site was surveyed only once, between August 2010 and August 2012.

**Table 1:** Summary of data collected in the Mesoamerican Reef System between 2010 and 2012.

Country	Depth range (m)	Reef sites Surveyed (n)	Sites with *A. palmata*	Transects Surveyed (n)	Transects with *A. palmata*
**Mexico**	≤5	9	3	56	9
	5.1–10	14	7	86	13
**Belize**	≤5	22	3	132	6
	5.1–10	14	1	83	2
**Guatemala**	≤5	–	–	–	–
	5.1–10	1	0	8	0
**Honduras**	≤5	16	2	118	5
	5.1–10	31	5	202	5
**Total**		**107**	**21**	**685**	**40**

The total number of sites and transects surveyed (total and with *Acropora palmata*) are shown for two depth ranges per country: equal to or less than 5 m (≤5) and from 5.1 to 10 m.

At each reef site, between four and ten 10 m line transects, separated by a least 5 m, were haphazardly deployed on top of the reef, parallel to the coast. At each site, the cover of all benthic components in the transects (including *A. palmata*) was measured by means of the line-point counts method, following the AGRRA protocol for benthic components [32]. A surveyor recorded the benthic component intercepting the line each 10 cm (*i.e.,* 100 points per transect). The cover of *A. palmata* was estimated as a percentage of the number of points overlaying *A. palmata* to the total number of points. All surveys were conducted by SCUBA.

### Historical Data of the Northern Portion of the Mesoamerican Reef System

Estimates of the cover and distribution of *A. palmata* for the coast of Quintana Roo, Mexico for 1985 were obtained from the Atlas of Mexican Caribbean Reefs [35] and from one unpublished report [36]. The data in these two studies were collected by the same surveyor (E. Jordán-Dahlgren), and with the same methodology. At each reef site, five 20 m long line-transects were haphazardly deployed perpendicular to the coast. Transects were separated from each other by at least 5 m. *Acropora palmata* cover was measured *in situ* by recording the number of centimeters along the transect that overlaid this species (the line-intercept method [37]). The percentage of *A. palmata* cover was then calculated by averaging the five transects. Because our aim was to compare the cover and distribution of *A. palmata* with the most recent data (see previous section), we selected only reef sites ≤10 m deep that were in geographically similar localities, although not exactly in the same, precise location, to those surveyed in 2010–2012 (Fig. S1). In total 31 reef sites were selected.

Unfortunately, the original databases for these studies were lost (Jordán-Dahlgren, pers. comm.). Therefore, we scanned each relevant figure (bar-plots) from the Atlas of Mexican Caribbean Reefs [35] and the unpublished report [36], and extracted the *A. palmata* mean cover estimates for each site with the help of SigmaScan Pro software version 4.0. Since these data were obtained from scanned figures (instead of raw data) these values should be considered as ‘best estimates’.

#### Limitations of the Study

Some limitations of the study must be acknowledged. There were differences in the methodologies used to estimate *A. palmata* cover in 1985 and 2010–2012, however, the two methods employed are known to produce relatively accurate and similar estimates of benthic cover [38]. Also, the reef sites surveyed at both periods of time were not the same exact geographical location and given the high variability in local coral community structure and local reef dynamics the results cannot be extrapolated to the whole region. Reef morphology varies throughout the MRS and we could not make geographical comparisons of *A. palmata* cover while holding constant factors such as zone, depth and wave exposure.

### Statistical Analyses

Violations of assumptions of normality, homoscedasticity and outliers in the dataset precluded the use of parametric tests. The interquartile range (from here on referred to as IQR) is the difference between the first and third quartiles of the data (or the 25^th^ and 75^th^ percentiles) and the outliers here refer to data points beyond this range. The R program version 2.15.3 [39] was used for statistical analyses, using the packages pgirmess and nparcomp. Non-parametric ANOVAs were conducted based on the Kruskal-Wallis rank procedure (kruskal.test) and nonparametric multiple comparisons for relative contrast effects were performed with nparcomp using Tukey’s adjustment [40]. In all analyses *α* = 0.05.

## Results

### Mesoamerican Reef System Regional Survey (2010–2012)

In 2010–2012, *A. palmata* was recorded in 21 of the 107 reef sites surveyed along the MRS, ten of these reef sites were in Mexico (out of 23), seven in Honduras (out of 47), and four in Belize (out of 36); *A. palmata* was not recorded in the only site surveyed in Guatemala (Fig. 1a). Overall, the occurrence of *A. palmata* was only recorded in 5.8% of the 685 transects carried out in the MRS, with the northern section of the region (*i.e.,* in the northern part of the Mexican Caribbean) showing a relatively higher occurrence of this species: 55% of the transects with *A. palmata* were recorded in Mexico, 20% in Belize and 25% in Honduras (Tables 1 and S1).

The cover of *A. palmata* in the 21 reef sites where this species was recorded was low and patchy (Table S1), with an overall mean value of 4.0% (SD: 10.1%) and an overall median value of 0.0% (IQR, 0.0% to 1.0%). Only in three sites was *A. palmata* recorded in more than 50% of the transects and had a median percentage of cover higher than zero (Fig. 1b). Two of these sites are located in the northernmost section of the MRS (La Bandera and Limones reefs, in Mexico) and one in Belize (Cay Caulker) (Fig. 1b). The median *A. palmata* cover was statistically different between the 21 reef sites (Kruskal-Wallis test H_(20, N = 138)_ = 34.35, p = 0.0239). A post-hoc nonparametric multiple test procedure showed that the *A. palmata* cover in the Limones reef site (Mx 2) was significantly higher (p<0.001) than that of all other reef sites, except for Punta Venado (Mx3), Akumal (Mx5 and Mx6), Tulsayab (Mx7), San Pedro (Be1), Cay Caulker (Be2), Roatan (Ho2), Salmedina’s Cay (Ho6) and Cocalito (Ho7). Although the median *A. palmata* cover in the Limones reef site was considerably higher than the other sites, the multiple comparison test did not detect differences due to the high variability among transects within sites (Fig. 1b). No significant differences (p>0.05) were found between any other pair of reef sites.

#### Limones reef site, Mexico

**Figure 2:**
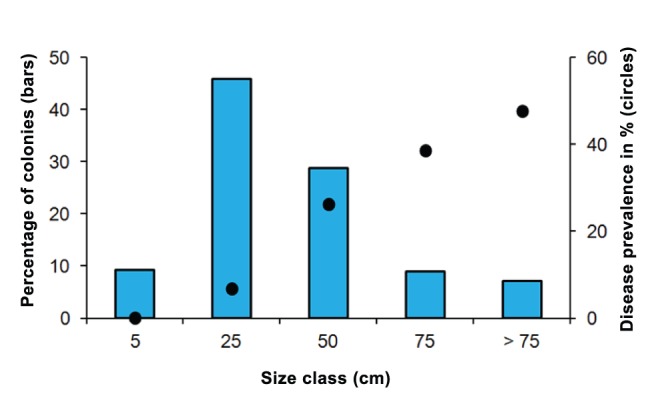
Size frequency distribution (blue bars) of *Acropora palmata* colonies at Limones reef, Mexico in 2012 (n = 292 colonies). Black circles indicate disease prevalence.

Given the high *A. palmata* cover in the Limones reef site, we conducted a further survey to gain a better understanding of the population structure and condition of *A. palmata* at this site. Five 25×1 m transects were surveyed in the summer of 2012 to inspect for the presence of diseases and predators, and to estimate the percentage of total recent and old mortality in all the *A. palmata* colonies intercepted by each transect. Overall, the Limones reef site had an abundant and healthy population of *A. palmata.* We found on average 2.3 (SD = 1.6; range 0.8–5.0) colonies m^-2^, with a size frequency distribution skewed towards small (<50 cm in diameter) colony sizes (Fig. 2). White-band disease was observed in only 1% of the colonies. However, 14.7% of the colonies had irregular-shaped white lesions resembling white-pox disease [14]. The percentage of colonies affected by diseases increased with size; disease prevalence was zero percent in the 5 cm size class, whereas in colonies larger than 75 cm it rose to 48% (Fig. 2). Common predators of *A. palmata* (*i.e., Coralliophila abbreviata*, *Hermodice carunculata* and damselfishes) were observed on only 0.3% of the colonies. The majority of *A. palmata* colonies were completely covered by live tissue: 72.3% of the colonies had no partial mortality, 21.2% had less than 25% mortality and only 6.5% had more than 25% mortality.

### 25 years of Change in *Acropora palmata* in Mexico’s Portion of the MRS

**Figure 3:**
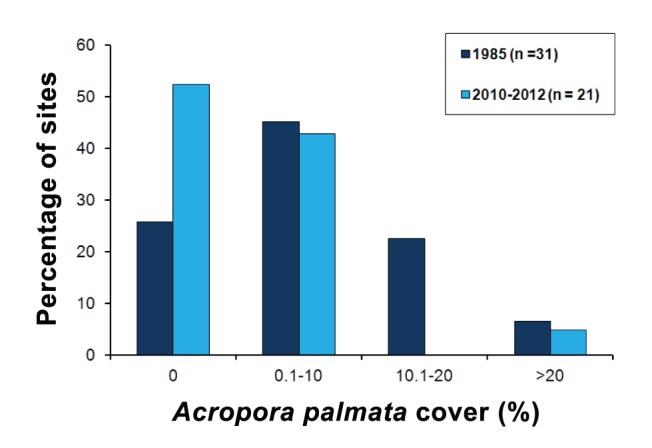
Frequency distributions of mean *A. palmata* cover recorded in the Mexican portion of the MRS in 1985 (dark blue bars) and in this survey (2010–2012, light blue bars). Data for 1985 are based on the study of Jordán-Dahlgren [35], [36]; n = number of sites.

The comparison of the distribution and cover of *A. palmata* in the Mexican portion of the MRS between the historical dataset (1985) and the most current estimates (2010–2012), showed a decline in the abundance of this species. In 1985, *A. palmata* was recorded in 74% of the sites that were surveyed, while in 2010–2012 it was recorded in only 48%. Overall, the mean *A. palmata* cover decreased from 7.7% (SD = 9.0) in 1985 to 2.9% (SD = 7.5) in 2010–2012. The frequency distribution of *A. palmata* cover in this region also showed a severe decrease in the number of reef sites containing relatively high cover of this species (10–20%; Fig. 3). Interestingly, the reef site with the highest *A. palmata* cover in 1985 (mean = 37.2%) is located 1.5 km south of the Limones reef site.

## Discussion

The distribution and abundance of *Acropora palmata* is limited throughout the Mesoamerican Reef. Our findings show that in the 2010–2012 surveys, this species was present only in 20% of the 107 surveyed sites, and that it was abundant only in the Limones reef site (mean = 34.7%, SD = 24.5). While, the presence of this reef site is encouraging, the regional picture suggests that *A. palmata* is failing to recover along the MRS. In the Mexican coast of the MRS, the percentage of sites with *A. palmata* decreased from 74% to 48%, between 1985 and 2010–2012, and the overall mean cover declined from 7.7% (SD = 9.0) to 2.9% (SD = 7.5). Although we were not able to make historical comparisons for Belize and Honduras, the low levels of *A. palmata* documented in both countries in 2010–2012 suggest a similar situation for the entire MRS.

The decline in *A. palmata* distribution and abundance that we describe for the Mexican Caribbean represents one of the first large-scale quantitative reports for the Caribbean, and is consistent with a recent Caribbean-wide study, which shows that the proportion of locations with *A. palmata* presence has rapidly declined from the 1970s to the present [41]. Local-scale studies also report similar trends in recent decades. In Glover Reef, Belize, McClanahan et al. (1998) measured a 99% loss in *A. palmata* cover between 1970 and 1997 [18]. For Looe Key, Florida, Miller et al. (2002) estimated that *A. palmata* declined by 93% between 1983 and 2000 [19]. In the present study it was not possible to further explore changes at site level because the same sites were not surveyed in the two time periods (1985 vs. 2010–2012). Nevertheless, the relatively low mean *A. palmata* cover reported for Mexican reefs in 1985 (7.7%) suggests that the populations of this species were already deteriorated by that year. This is supported by some studies reporting that *A. palmata* cover values above 25% were common on shallow reefs of the northeastern Yucatan Peninsula during the late 1970s [35], [36], [42].

Conservation and management actions during the last 20 years have not sufficed to prevent the continued decline of *A. palmata* in the MRS. There are at least three non-exclusive reasons that could explain this situation. First, the 1980s widespread mortality of *A. palmata* could have drastically reduced the viable populations of this species to such an extent that the connectivity between populations was affected. As a consequence, the genetic diversity of this species would decrease considerably and negatively affect its recovery [43]. Second, although local rehabilitation efforts have been conducted, no ecosystem-wide restoration projects aiming to facilitate the large-scale recovery of *A. palmata* have been implemented in the MRS. Local rehabilitation efforts usually consist of fixing detached fragments of *A. palmata* following the impact of a hurricane or other physical damage [21]. These efforts have proven to be successful in some cases, but usually are short-term (1–3 years) and spatially limited [21], and thus are unlikely to increase *A. palmata* distribution and abundance on a regional scale. Lastly, the populations of *A. palmata* (and other coral species) are still affected by other regional and global stressors, such as diseases, bleaching episodes and land-based threats. For example, in the MRS there still exists the need for an integrated coastal-zone management plan that includes programs to actively reduce the impact of watershed pollution and other inland threats [44]–[46]; and globally more proactive agreements and legislations are needed to reduce the negative consequences of global climate change [22], [47].

**Figure 4:**
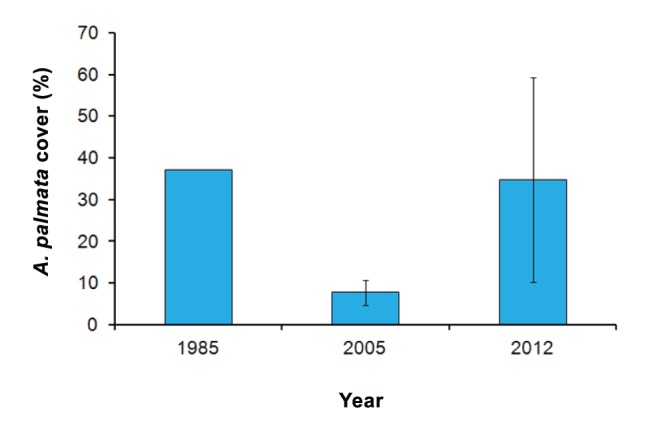
Mean percent *Acropora palmata* cover (± SD) in Limones reef site in 1985, 2005 and 2012. Data for 2005 were taken by the Puerto Morelos Reef National Park staff in four 30-m permanent transects in November 2005 after the impact of Hurricanes Emily (July) and Wilma (October). Only the mean value is reported for 1985.

The Limones reef site has an outstanding ecological and conservational value. Data obtained from other studies on this reef (although not in the exact same location) suggest that the populations of *A. palmata* are highly resilient. In the fall of 2005, after the impact of two major Hurricanes (Emily and Wilma), which affected the entire northeastern Yucatan coast [48], *A. palmata* cover dropped to less than 10%, according to the monitoring data of the Puerto Morelos Reef National Park (PMRNP; Fig. 4). In this study we report that the cover of *A. palmata* by mid-2012 was above 30%, a very similar figure to what was reported in the late-1970s for northeastern Yucatan reefs [35], [36]. The increment in *A. palmata* cover at the Limones reef site might have resulted, at least in part, from a rehabilitation effort, conducted by the PMRNP one month after the hurricanes of 2005, when 221 fragments, with a mean size of 31 cm (SD = 16 cm; PMRNP *unpubl. data.*), were relocated between the back-reef and the reef-crest of the Limones reef site. Unfortunately, there was no follow-up to this effort, so it remains unknown how many fragments survived. The high proportion of small colonies and the low partial mortality recorded in the present study (Fig. 2), suggests that larval recruitment at a later date has also contributed to the recovery of *A. palmata* in the Limones reef site. The local oceanographic conditions at this site favor the formation of eddies [49] that may tend to promote larval retention and accumulation [50]. Nevertheless, it is evident that more detailed studies are needed in order to understand the ecological and environmental conditions that have favored the persistence of a healthy population of *A. palmata* in this reef site. In addition, a description of the genetic diversity of the *A. palmata* stands from this site would contribute towards the development of novel conservation and propagation strategies for this species.

Determining the genetic variability of *A. palmata* populations in the few reef sites with healthy and abundant *A. palmata* populations is crucial to identifying whether there are resistant genotypes that make certain populations more resistant to diseases. Vollmer and Kline [51] showed that six percent (3 out of 49) of *Acropora cervicornis* genotypes in Bocas del Toro, Panama, were resistant to white-band disease, and suggested that resistant genotypes may explain why pockets of *Acropora* have been able to survive the white-band disease epidemic for three decades. These authors suggest that because *A. cervicornis* and *A. palmata* have a close evolutionary relationship, it is likely that white-band disease resistance exists in *A. palmata* as well. Genetic surveys for resistance genes to white-band and white-pox diseases in *A. palmata* should be therefore a research priority. Restoration programs should be designed to also include sexual recruits, rather than depending only on fragments, in order to maintain genetic diversity. More extensive surveys along the MRS could help to identify other reef sites with relatively high *A. palmata* cover, and population genetics surveys on these sites would contribute to increase our understanding of the genetic diversity of these populations. Care should be taken, however, in the development of rehabilitation programs that use sexual recruits without an adequate understanding of the population genetic structure. In particular, transplanting genotypes that are potentially more susceptible to diseases or other stressors to other reefs should be avoided in order to prevent a negative effect on the abundance of the populations. Therefore, emphasis should also be placed on determining the susceptibility of *A. palmata* populations to different diseases and global change stressors.

Our findings show that, despite conservation and management actions over the last 20 years, *Acropora palmata* populations along the MRS are fragile, with low recovery after the mass mortality events that occurred in the 1980s. Long term restoration and propagation projects, using both fragments and sexual recruits with disease resistant genotypes, need to be conducted to determine if they could help the recovery of *A. palmata* populations along the MRS. Reefs with healthy populations of *A. palmata* should also be established as priority areas for research, to identify the biological, physiological, ecological and physical oceanographic factors that could be playing a role in the higher *A. palmata* cover. Developing special management considerations for the protection of the few sites with relatively high *A. palmata* cover along the MRS should be a priority (Fig. 1; Table 1), this might include the designation of critical habitats and developing integrated coastal-zone management plans to reduce inland threats [44]–[46]. In September 2013, and partly because of the findings of this study, the Puerto Morelos Reef National Park authorities decreed Limones Reef as critical habitat for *A. palmata*, and restricted tourism and fishing activities in the area. To our knowledge no other critical habitats exist for *A. palmata* in the MRS or elsewhere in the Caribbean, except for a few reefs in US territory [52].

## Supporting Information

Figure S1 **Location of the reef sites surveyed in 1985 and 2010–12 along the Mexican Caribbean coast.**
(DOCX)

Table S1 **Sites where** ***Acropora palmata*** **was recorded along the Mesoamerican Reef in 2010–2012.**
(DOCX)
