# Supplementary material for: Assessment of Acropora palmata in the Mesoamerican Reef System
Source: PLoS One. 2014 Apr 24;9(4):e96140. doi: 10.1371/journal.pone.0096140 (PMC3999099; doi:10.1371/journal.pone.0096140)
Supplement: Figure S1 — Location of the reef sites surveyed in 1985 and 2010–12 along the Mexican Caribbean coast. (DOCX) [file pone.0096140.s001.docx]

Figure S1.

Figure S1. Location of the reef sites surveyed in 1985 (green triangles) and 2010-12 (red circles) along the Mexican Caribbean coast. The spatial coverage was extensive in both sampling periods but surveyed sites were not in the same precise geographical location. In 2010-12, the reefs between Punta Allen and Xcalak towns were not surveyed for logistical reasons, so even though some of these sites were surveyed in 1985 they were not included in the historical analysis.
