# Supplementary material for: Assessment of Acropora palmata in the Mesoamerican Reef System
Source: PLoS One. 2014 Apr 24;9(4):e96140. doi: 10.1371/journal.pone.0096140 (PMC3999099; doi:10.1371/journal.pone.0096140)
Supplement: Table S1 — Sites where Acropora palmata was recorded along the Mesoamerican Reef in 2010–2012. (DOCX) [file pone.0096140.s002.docx]

**Table S1**. Sites (n = 21) where *Acropora palmata* was recorded along the Mesoamerican Reef in 2010-2012. Sites are arranged from north to south for Mexico and Belize and from West to East in Honduras. SD: Standard deviation.

| Country | Site code | Reef name | Date of survey | Latitude | Longitude | Depth | Transects (N) | | *A. palmata* Mean cover (SD) | *A. palmata* Median cover |
| --- | --- | --- | --- | --- | --- | --- | --- | --- | --- | --- |
|  |  |  |  |  |  |  | **Surveyed** | **w/ *A. palmata*** |  |  |
| Mexico | Mx1 | La Bandera | 24/10/2011 | 21.1705 | -86.7297 | 6.5 | 6 | 4 | 3.2 (4.2) | 1.0 |
| Mexico | Mx2 | Limones | 26/03/2012 | 20.9872 | -86.7964 | 1.5 | 6 | 6 | 34.7 (24.5) | 28.0 |
| Mexico | Mx3 | Punta Venado | 17/05/2012 | 20.5509 | -87.1492 | 3.9 | 6 | 2 | 6.8 (11.6) | 0.0 |
| Mexico | Mx4 | Yalku | 04/05/2102 | 20.4074 | -87.2995 | 6.0 | 7 | 2 | 0.4 (0.8) | 0.0 |
| Mexico | Mx5 | Akumal 1 | 20/06/2011 | 20.3838 | -87.3158 | 7.6 | 6 | 1 | 2.3 (5.7) | 0.0 |
| Mexico | Mx6 | Akumal 2 | 20/06/2012 | 20.3833 | -87.3151 | 5.1 | 6 | 2 | 6.7 (13.2) | 0.0 |
| Mexico | Mx7 | Tulsayab | 19/09/2011 | 20.2506 | -87.3954 | 6.8 | 6 | 2 | 3.5 (6.4) | 0.0 |
| Mexico | Mx8 | Xcalak 1 | 01/04/2012 | 18.2401 | -87.8262 | 6.7 | 6 | 1 | 1.7 (4.1) | 0.0 |
| Mexico | Mx9 | Xcalak 2 | 01/04/2012 | 18.2137 | -87.8274 | 9.8 | 6 | 1 | 1.5 (3.7) | 0.0 |
| Mexico | Mx10 | Xcalak 3 | 13/04/2012 | 18.1860 | -87.8330 | 1.5 | 6 | 1 | 0.2 (0.4) | 0.0 |
| Belize | Be1 | San Pedro | 25/08/2011 | 17.8817 | -87.9694 | 1.2 | 6 | 2 | 4.5 (7.1) | 0.0 |
| Belize | Be2 | Cay Caulker | 26/08/2012 | 17.7996 | -87.9954 | 1.3 | 6 | 3 | 5.5 (6.6) | 4.0 |
| Belize | Be3 | Lighthouse Reef | 06/12/2011 | 17.2591 | -87.5558 | 1.6 | 6 | 1 | 0.7 (1.6) | 0.0 |
| Belize | Be4 | Glover Reef | 23/11/2011 | 16.7200 | -87.8387 | 9.0 | 6 | 2 | 1.8 (4.0) | 0.0 |
| Honduras | Ho1 | Big Swan Channel | 19/07/2011 | 17.4104 | -83.9162 | 6.8 | 6 | 3 | 0.5 (1.2) | 0.0 |
| Honduras | Ho2 | Roatan 1 | 21/07/2011 | 16.3982 | -86.2824 | 7.7 | 6 | 2 | 6.3 (11.7) | 0.0 |
| Honduras | Ho3 | Roatan 2 | 01/09/2011 | 16.4071 | -86.4071 | 4.8 | 7 | 1 | 0.1 (0.4) | 0.0 |
| Honduras | Ho4 | Cayo Cochinos | 30/08/2010 | 15.9811 | -86.4787 | 5.4 | 8 | 1 | 1.0 (2.8) | 0.0 |
| Honduras | Ho5 | Utila | 14/12/2011 | 16.1215 | -86.9151 | 9.3 | 6 | 1 | 0.2 (0.4) | 0.0 |
| Honduras | Ho6 | Salmedina's Cay | 21/04/2012 | 16.0430 | -86.9797 | 7.6 | 10 | 1 | 1.4 (4.4) | 0.0 |
| Honduras | Ho7 | Cocalito | 28/05/2011 | 15.9118 | -87.6171 | 3.1 | 10 | 3 | 5.0 (9.1) | 0.0 |
